# A High-Precision Plant Disease Detection Method Based on a Dynamic Pruning Gate Friendly to Low-Computing Platforms

**DOI:** 10.3390/plants12112073

**Published:** 2023-05-23

**Authors:** Yufei Liu, Jingxin Liu, Wei Cheng, Zizhi Chen, Junyu Zhou, Haolan Cheng, Chunli Lv

**Affiliations:** 1 College of Information and Electrical Engineering, China Agricultural University, Beijing 100083, China; 2 College of Economics and Management, China Agricultural University, Beijing 100083, China; 3 International College Beijing, China Agricultural University, Beijing 100083, China

**Keywords:** dynamic pruning, low-computing-platform friendly, re-parameterization, deep learning

## Abstract

**Simple Summary:**

Achieving automatic detection of plant diseases in real agricultural scenarios where low-computing-power platforms are deployed is a significant research topic. As fine-grained agriculture continues to expand and farming methods deepen, traditional manual detection methods demand a high labor intensity. In recent years, the rapid advancement of computer network vision has greatly enhanced the computer-processing capabilities for pattern recognition problems across various industries. Consequently, a deep neural network based on an automatic pruning mechanism is proposed to enable high-accuracy plant disease detection even under limited computational power. Furthermore, an application is developed based on this method to expedite the translation of theoretical results into practical application scenarios.

**Abstract:**

Timely and accurate detection of plant diseases is a crucial research topic. A dynamic-pruning-based method for automatic detection of plant diseases in low-computing situations is proposed. The main contributions of this research work include the following: (1) the collection of datasets for four crops with a total of 12 diseases over a three-year history; (2) the proposition of a re-parameterization method to improve the boosting accuracy of convolutional neural networks; (3) the introduction of a dynamic pruning gate to dynamically control the network structure, enabling operation on hardware platforms with widely varying computational power; (4) the implementation of the theoretical model based on this paper and the development of the associated application. Experimental results demonstrate that the model can run on various computing platforms, including high-performance GPU platforms and low-power mobile terminal platforms, with an inference speed of 58 FPS, outperforming other mainstream models. In terms of model accuracy, subclasses with a low detection accuracy are enhanced through data augmentation and validated by ablation experiments. The model ultimately achieves an accuracy of 0.94.

## 1. Introduction

The most significant challenges that any crop faces are diseases [1], pests [2], weeds [3], and nutritional deficiencies. Among them, identifying plant diseases through an optical analysis of disease signs on plant leaves presents a significant challenge. Farmers and domain experts used manual methods for detecting disorders by visualizing the plant’s leaf with the naked eye. However, this method has become infeasible due to the large size of fields, physical conditions, time, and cost. Furthermore, due to the variety of cultivated plants and the range of phytopathological issues they can encounter, there is an increased risk of inaccurate diagnosis and treatment [4]. Therefore, automatic, robust, precise, fast, and cost-effective methods and techniques for plant disorder identification have been demanding research in smart agriculture in recent years.

In [5], the author focused solely on a particular type of tomato leaf image and employed a CNN for disease classification; they utilized LVQ as the network classifier, achieving an 86% accuracy rate on the test set. However, studying only a single plant does not reflect the model’s generalizability. Therefore, in 2016, Mohanty et al. [6] expanded their research to 14 crops and 26 diseases. The trained model achieved a 99.35% accuracy rate on the reserved test set, demonstrating the feasibility and some universality of deep learning in crop disease detection. To further improve plant disease detection techniques, Xu et al. [7] provided an approach for data augmentation that optimized the model by utilizing nontarget area data in sample images. Inspired by this approach, Zhang et al. [8] extended deep convolutional generative adversarial networks (DCGAN) for detecting defects in pear images. The results showed that that enhanced CNN’s performance was significant, with a detection accuracy rate of 97.35% on a validation set of 3000 images. Building on the above models, Yasamin Borhani et al. [9] trained a total of five CNN models, including VGG16, ResNet-50, Inception, MobileNet-V3, and EfficientNet-B0, and performed classification. The results demonstrated that EfficientNet-B0 had the highest accuracy rate in low-cost computation situations. This provided a new perspective for the further exploration of CNNs.

Apart from CNNs, the methods commonly used to tackle this task include YOLO and Transformer [10,11,12]. Liu and Wang [13] improved the existing tomato disease image recognition technology based on the YOLOv3 model, achieving application transfer. However, YOLOv3 is not a panacea, as it still has some of its features that do not work, such as anchor box x, y, offset predictions, linear x, y predictions instead of logistic ones, a focal loss, dual IOU (intersection over union) thresholds, and the ground-truth assignment. To overcome the above drawbacks, Midhun P. Mathew et al. [14] achieved a lightweight and efficient plant disease detection by implementing it on smartphones based on YOLOv5. The latest trend is to use the attention mechanism to improve the performance of plant disease detection models. A typical example is the introduction of the attention mechanism into a residual CNN for tomato disease detection by Karthik et al. [15], who achieved a 98% accuracy on a dataset containing 95,999 tomato leaf images. Lu et al. [16] went even further by combining GhostNet and ViT to design a novel model. That model achieved a 98.14% accuracy when evaluating 11 classes of grape leaf images totaling 12,615 on the GLDP12k dataset. Despite achieving a high precision, the interpretability of these models has yet to be explored. To address this issue, Poornima Singh Thakur et al. [17] proposed an advanced model called “PlantXViT”, which not only ensured a high accuracy but also revealed the essence of plant diseases to a certain extent.

Despite the significant progress made in using deep learning for detecting plant diseases, various challenges still affect the reliability and performance of the technique, making it extremely challenging to identify plant-specific diseases using deep learning:

1. Acquiring relevant datasets of plant leaf images for specific diseases is a challenging task. Only a limited number of studies [18,19,20] have utilized sizable datasets consisting of thousands of images or more. Moreover, the high costs associated with hardware make it challenging to deploy models on mobile devices.
2. On a single leaf, there may coexist various distinct maladies, while the resemblance among infection areas can prompt researchers to extract improper characteristics, leading to an erroneous categorization based on unrelated features [21].
3. The deep neural networks represented by CNNs often encounter the issues of overfitting or excessive training, which must be overcome. Furthermore, the model’s generalization ability is not satisfactory, and there is an urgent need to develop a model that is generally effective for different plant leaves [22,23].
4. The inference speed of the model is relatively slow, making it difficult to adapt to actual production processes [24,25,26].

To address the aforementioned challenges and enhance the efficiency of plant disease detection, this paper proposes a high-precision plant disease detection method that requires only a low computing power. The main contributions and innovations of this paper are as follows:

1. A re-parameterization method is proposed to improve the boosting accuracy of convolutional neural networks.
2. A dynamic pruning gate is introduced to dynamically control the network structure, enabling operation on hardware platforms with significant differences in computing power.
3. The theoretical model based on this paper is implemented, and the development of the application program is completed.

## 2. Related Works

### 2.1. Convolutional Neural Network (CNN)

A CNN exhibits robust feature learning capabilities, which have proven to be remarkably effective in the detection of plant diseases, thus garnering considerable favor among researchers. Mohanty et al. [6] were the first to utilize a CNN for the detection of plant diseases on a large-scale dataset. Through a performance comparison between AlexNet and GoogleNet, it was found that the GoogleNet model with transfer learning achieved a precision of 99.35% on the PlantVillage dataset. Following this, Thakur et al. [27] developed a CNN that used two pretrained VGG16 and Inception v7 layers. They expanded upon the PlantVillage dataset with an additional four datasets, including the Embrapa, Apple, Maize, and Maize datasets. The results indicated a precision of 99.16%, 93.66%, 94.24%, 91.36%, and 96.67% on the five datasets, respectively, further validating the potential and universality of CNNs in this field. However, considering a CNN’s expertise in extracting local features and its difficulty in capturing global clues, Jiang et al. [28] improved a CNN by introducing the Inception structure and Rainbow concatenation from GoogleNet and training the resulting INAR-SSD (SSD with Inception module and Rainbow concatenation) model to detect five common apple leaf diseases. Experimental results showed that the INAR-SSD model achieved a high detection speed of 23.13 FPS and a mAP of 78.80%.

Xu et al. [7] proposed a data augmentation technique to further improve plant disease detection technology. Their method utilized the prior mask as input and effectively optimized the model by leveraging nontarget region data in sample images. Inspired by similar ideas, Zhang et al. [8] used an enhanced CNN to detect defects in pears, specifically by extending defect images through deep convolutional generative adversarial networks (DCGANs). The results demonstrated significant improvements with the enhanced CNN achieving a detection accuracy of 97.35% on a validation set of 3000 images. Building on these models, Yasamin Borhani et al. [9] trained a total of five CNN models for classification, including VGG16, ResNet-50, Inception, MobileNet-V3, and EfficientNet-B0. The results indicated that EfficientNet-B0 had the highest accuracy at a low computational cost. This offered a new perspective for further exploration of CNNs.

In addition to classic architectures such as AlexNet, GoogleNet, VGG16, and ResNet, which utilize transfer learning, there are also studies that have introduced customized CNN architectures for plant disease detection tasks [29,30]. These studies have greatly expanded the application range of CNNs in this field.

### 2.2. You Only Look Once (YOLO)

The detection of diseases in plant leaves can be considered as an object detection problem [31]. Therefore, one of the commonly used methods to solve this task is YOLO. Arsenovic et al. [32] proposed a PlantDiseasenet network composed of two levels of structure: PDNet-1 and PDNet-2. PDNet-1 employed the YOLO to detect plant leaves, while PDNet-2 was responsible for leaf classification. After being trained, that model achieved an accuracy of 93.67%.

In 2018, Joseph Redmon proposed a YOLO model called YOLOv3: An Incremental Improvement in his paper [33], which outperformed YOLOv2. YOLOv3 achieved three times the accuracy of traditional networks. Subsequently, the introduction of YOLOv3 in the field of research has become widespread. Liu and Wang [13] optimized the feature layer of the YOLOv3 model using image pyramids, enabling multiscale feature detection and improving both the detection accuracy and speed of the YOLOv3 model. Meanwhile, Tian et al. [34] utilized DenseNet to optimize the feature layer of a low-resolution YOLOv3 model from different perspectives, improving the detection of apple anthracnose disease damage by the YOLOv3 model. These studies have all demonstrated the excellent potential of YOLOv3 in agricultural detection tasks. YOLOv4, which is an improved version of YOLOv3, generated bounding-box coordinates and assigned probabilities to each category, converting the object detection task into a regression problem. Apu Shill et al. [35] conducted comparative experiments on the PlantDoc dataset and found that the overall performance of YOLOv4 was better than YOLOv3, with an average precision increase of approximately 2.37%. Since then, the use of YOLOv4 in plant disease detection research has become increasingly prevalent. More typical is the improved version of YOLOv4 proposed by Rikhi Bose et al. [36]. The modified network architecture maximized both detection accuracy and speed by including DenseNet in the backbone of the network to optimize feature transfer and reuse, and two new residual blocks in the backbone and neck enhanced feature extraction and reduced computing cost; the spatial pyramid pooling (SPP) enhanced the receptive field, and a modified path aggregation network (PANet) preserved the fine-grain localized information and improved feature fusion. Multiple improvements resulted in an accuracy of 90.33% for the model at a detection rate of 70.19 FPS.

However, YOLOv3 and YOLOv4 are not infallible. Some of their features do not work, such as anchor box x, y, offset predictions, linear x, y predictions rather than logistic ones, a focal loss, dual IOU (intersection over union) thresholds, and the ground-truth assignment [14]. To overcome these shortcomings, Midhun P. Mathew et al. [14] developed a model based on YOLOv5, which detected bacterial spot disease in bell pepper plants using a mobile device as a carrier. With the same GPU and dataset, the training time of that model was only 9.5% of that using the YOLOv4 model, achieving the goals of a lightweight and efficient performance.

### 2.3. Transformer

Apart from the various model architectures mentioned above, the latest trend is to utilize attention mechanisms to enhance the performance of plant disease detection models. In attention mechanisms, pixel locations with relevant information are given higher priority, which effectively compensates for the drawback of CNNs in capturing global clues [37]. Inspired by this, researchers have effectively utilized attention mechanisms to improve the classification performance of CNNs. Karthik et al. [15] developed a residual CNN with attention mechanisms for tomato disease detection. The model achieved an accuracy of 98% on a dataset containing 95,999 tomato leaf images, demonstrating a remarkable performance. In addition, Zhao et al. [38] developed a CNN with inception modules and residual blocks using an improved convolutional block attention module, achieving an accuracy of 99.55% on corn, tomato, and potato datasets. The transfer application of the attention mechanism goes far beyond these studies. Chen et al. [39] used spatial and channelwise attention modules with depthwise separable convolution in DenseNet. The model performed well on the maize variety in the PlantVillage dataset, achieving an accuracy of 98.50%, and achieving an accuracy of 95.86% on the Maize dataset as well. In another work by Chen et al. [40], spatial and channelwise attention mechanisms were also applied to MobileNet, which achieved excellent classification performance on the Rice dataset with an accuracy of 98.48%.

Although the above deep learning techniques are very promising, most of them either have high requirements for memory and computing power or a limited model generalization due to their lightweight design. To address these issues, Pritee Khanna et al. [41] proposed a plant disease detection model “PlantViT” based on Transformer. The model achieved accuracies of 98.61% and 87.87% on the PlantVillage and Embrapa datasets, respectively, effectively constructing a lightweight and compact plant disease detection model. Lu et al.’s [16] research also falls into the same category. They combined GhostNet and ViT and evaluated 12,615 grape leaf images from 11 categories on the GLDP12k dataset, achieving an accuracy of up to 98.14%. To further explore the interpretability of the model, Poornima Singh Thakur et al. [17] proposed a model called “PlantXViT”. The model consisted of the initial two blocks of the pretrained VGG16 network, followed by an inception block and four stacks of transformer encoders. While ensuring a high accuracy, it also revealed the essence of plant diseases to some extent.

## 3. Results

### 3.1. Experiment Settings

All experiments on the dataset in the paper were repeated three times using a different training/testing split, and the results were finally averaged to ensure a stable conclusion. For each experiment, the dataset was randomly split into 50% for training and the other 50% for testing.

The network was trained using an SGD optimization with an initial learning rate of $1\times 10^{-4}$ and 500 epochs; the batch size was four, alternating inputs of positive and negative sequence pairs. For computational reasons, the whole sequence was not used during training, but 16 consecutive video frames were randomly sampled from the line.

### 3.2. Experiment Results

#### 3.2.1. Overall

In the experimental setup, all models were initially trained and subsequently evaluated using the validation set. Experimental results were acquired by averaging over several tests. Since the mAP metrics in the target detection task depended on different recalls, the recall under which the mAP was obtained is indicated in Table 1 in the form of mAP@recall. In the model comparison section, mainstream target detection models were selected: Faster RCNN, a representative of two-stage detection models, and SSD, YOLO series, representatives of one-stage detection models. The results are shown in Table 1.

From the table, it can be seen that the proposed model outperformed current mainstream detection models in terms of mAP@50, mAP@75, recall, and precision. The precision metric was 33% greater than the Faster RCNN model. This could be attributed to Faster RCNN having only a single feature extraction network, which could not effectively extract all the features of the image in the feature extraction stage. In contrast, the model generated by re-parameterization exhibited a significant advantage over other models in all indexes due to the feature extraction capability of multiple networks.

**Figure 1:**
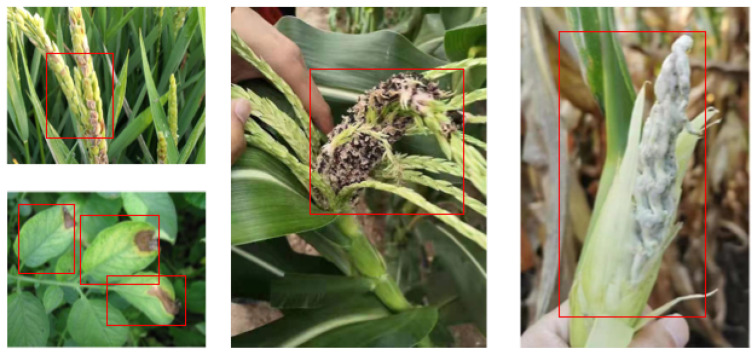
Recognition results based on our method. The red boxes are the bounding boxes given by our method.

To visualize the results mentioned above, the detection results of various diseases using the proposed method are presented in Figure 1.

From Figure 1, it can be seen that the proposed model not only led other models in terms of metric data but also had good performance in terms of actual detection results.

#### 3.2.2. Test on Different Devices

In general, the computing power of computing devices deployed in agricultural scenarios is much lower than that of GPU platforms. Therefore, in order to test the recognition speed of different models in different scenarios, we tested multiple models under four computing platforms. The computational power was ranked from highest to lowest, namely an RTX 3080 GPU with 12 GB of video memory, a PC laptop with 2060 GPU, a Jetson Nano with CUDA computational core dedicated to neural network inference, and a Huawei P40, a mobile platform for cell phones. The experimental results are shown in Table 2.

From Table 2, it can be observed that the dynamic pruning mechanism used in this study effectively improved the inference speed of the model on each computing platform. The inference speed of this model on a smartphone even exceeded that of Faster RCNN on an RTX 3080 GPU, a professional neural network accelerator. The fastest inference speed was achieved on all computing platforms. Notably, on very low power platforms, such as smartphones without a TPU, other models were unable to complete the inference task (due to the limitation of memory and CPU scheduling policy of smartphones), but the proposed model could still complete the inference process. This experimental result fully illustrates the effectiveness and robustness of the proposed approach.

#### 3.2.3. Test on Other Datasets

**Figure 2:**
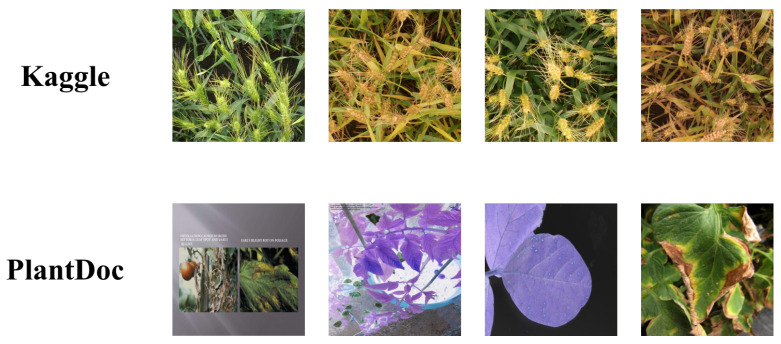
Samples of Kaggle and Plantdoc datasets.

To further verify the generalization of this study, open-source datasets on Kaggle [45] and Plantdoc [46] were used, which contained the image datasets as shown in Figure 2.

As can be seen from the figure, the differences between these two datasets are quite obvious. Kaggle uses a wheat cob labeling dataset with a resolution of 1024×1024; Plantdoc uses a plant disease dataset with a resolution of 416×416. Using these two datasets was a good way to verify the generalization performance of our model. The experimental results are shown in Table 3.

From Table 3, it can be seen that the proposed method could also achieve a 0.66 and 0.48 mAP on other open-source datasets on the Web, demonstrating the excellent generalization performance of the method.

### 3.3. Application on Mobile Platform

**Figure 3:**
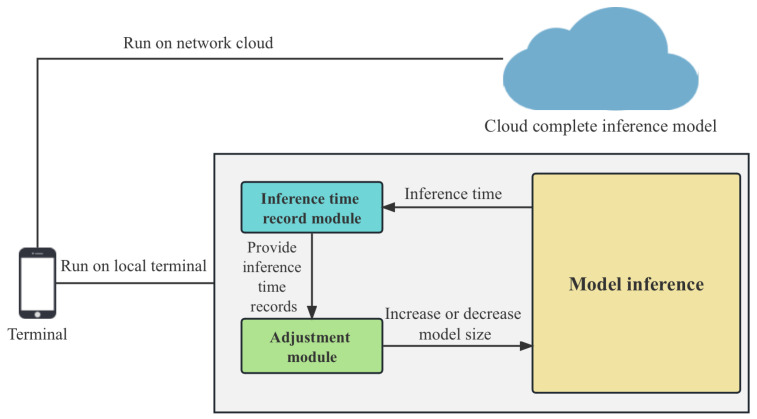
Illustration of client and server framework.

To quickly apply the model proposed in this study to farms, an application based on the WeChat platform was developed to package the model. Figure 3 shows the deployment process of the application.

**Figure 4:**
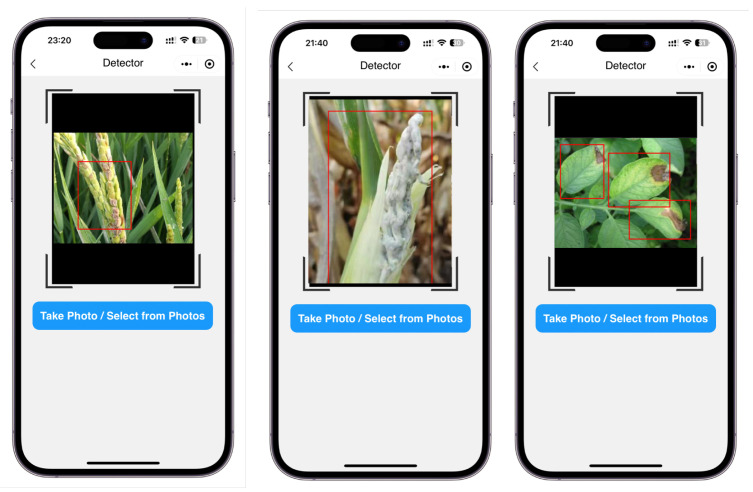
Screenshot of the detection effect running locally on the mobile terminal. Red boxes are the bounding boxes given by our method.

There were two specific deployment scenarios for mobile terminals: (1) the inference was performed locally at the terminal, as in Figure 3’s right branch and the detection screenshots shown in Figure 4; (2) the terminal only captured video streams and the inference was performed in the cloud, as in Figure 3’s upper branch. The experimental results of the first deployment scheme are shown in Table 2. In the second deployment scenario, the terminal compressed the captured video streams and sent them back to the server. The server ran the model proposed in this paper, recognized the video streams, and then sent the recognition results back to the mobile device for display.

## 4. Discussion

### 4.1. Comparison with Related Studies and Advantages of Our Method

In this section, the experimental results obtained in this study are discussed and compared with the results of other studies. The following is a comparison with the findings of other research studies.

Ref. [47] proposed a deep-learning-based method for wheat detection. Compared to our approach, their method achieved a similar performance in terms of mAP. However, the proposed method had an advantage in computational resource consumption, making it particularly suitable for platforms with low computational power. Ref. [48] explored the problem of strawberry fungal leaf disease detection using a convolutional neural network (CNN) approach. In comparison to our method, their method demonstrated an excellent accuracy. However, our method was more advantageous for reducing computational resource consumption. Ref. [49] investigated tomato classification using a deep-learning-based method. Although our method was slightly inferior in terms of mAP, it had a greater advantage in computational resource consumption. Ref. [50] discussed the issue of plant leaf disease detection. In comparison to our method, their method performed poorly in terms of mAP. On the contrary, our method achieved a better balance between accuracy and computational resource consumption. Ref. [51] focused on the impact of data augmentation on plant disease detection performance. Our study also experimented with different data augmentation methods and found that appropriate data augmentation techniques could enhance the performance of our method. Ref. [52] introduced a deep-learning-based rice plant disease detection method and tested it on multiple datasets. Our method had some similarity with this study in terms of generalization capability, but it was more advantageous in computational resource consumption. Ref. [53] addressed the challenges and future development of plant disease detection. Our research also recognized these challenges and explored and optimized computational resource consumption, data augmentation methods, and network structures. In future work, we will further focus on these challenges and seek solutions.

The field of deep learning is rapidly developing. To further validate the effectiveness of our method, we conducted experiments on multiple datasets and compared the proposed method with those of other researchers. Table 4 shows the comparison results of these different research efforts. Due to the nonreproducibility of some studies, we directly quoted the experimental results obtained in the original papers for other works. In the classification task involved in [24], a softmax classifier was concatenated with the backbone proposed in this paper, resulting in a classification network, and we performed experiments using this network to obtain the results.

By comparing the proposed method with the results of other studies, it can be found that the proposed method has the following advantages:

1. Higher accuracy: the proposed method achieved a higher accuracy in various detection and classification tasks, indicating that our method could more reliably detect plant diseases.
2. Lower computational resource consumption: the proposed method reduced the computational resource consumption through dynamic pruning gates, allowing it to run smoothly on platforms with low computational power, such as mobile devices.
3. Stronger generalization capability: the proposed method achieved a favorable performance on multiple plant disease datasets, indicating that it possessed strong generalization capabilities and could handle different types of detection tasks.
4. Exploration of data augmentation methods: the effects of different data augmentation methods on the performance of the proposed method were investigated in the experiments, and it was found that appropriate data augmentation techniques could enhance the performance of the method.

### 4.2. Limitation and Feature Works

In summary, through a comparison and discussion of the aforementioned literature, the proposed approach demonstrated certain advantages in the field of plant disease detection, particularly in achieving a better balance between accuracy and computational resource consumption. However, limitations and areas for improvement in this study are also recognized:

1. Detection performance for specific diseases may be limited: Although our method achieved a favorable performance across multiple datasets, it may still be suboptimal for certain specific disease detection tasks. In future research, we could design specialized network structures and training strategies for specific diseases to improve detection performance.
2. Optimization potential for computational resource consumption: While our method has already reduced computational resource consumption, it may still be unable to meet real-time detection requirements on some extremely low capability platforms. In future research, we can further explore more efficient network structures and pruning strategies to decrease computational resource consumption.
3. Exploration of data augmentation methods is still needed: Although various data augmentation methods were investigated in the experiments, many other data augmentation techniques remain unexplored. In future research, the effects of different data augmentation methods on plant disease detection performance can be further examined to identify more appropriate data augmentation strategies.

In conclusion, the present study has achieved some progress in the field of plant disease detection, but there are still many areas for improvement and challenges to overcome. In future research, the proposed approach will continue to be optimized to enhance the accuracy of plant disease detection, reduce computational resource consumption, and improve the generalization capability of the method. Meanwhile, collaboration with other researchers is also desired to jointly promote the development of the plant disease detection field.

## 5. Materials and Methods

### 5.1. Dataset Analysis

**Figure 5:**
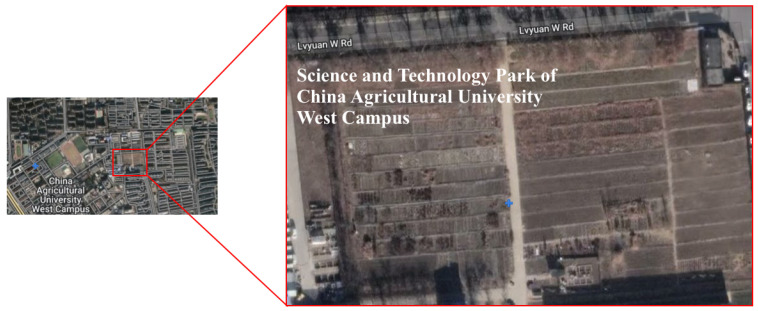
This figure presents the dataset collection sites (Science and Technology Park of the West Campus of China Agricultural University) on Google Map.

The dataset was collected from the Science and Technology Park of the West Campus of China Agricultural University, as shown in Figure 5, from October 2019 to February 2023.

**Figure 6:**
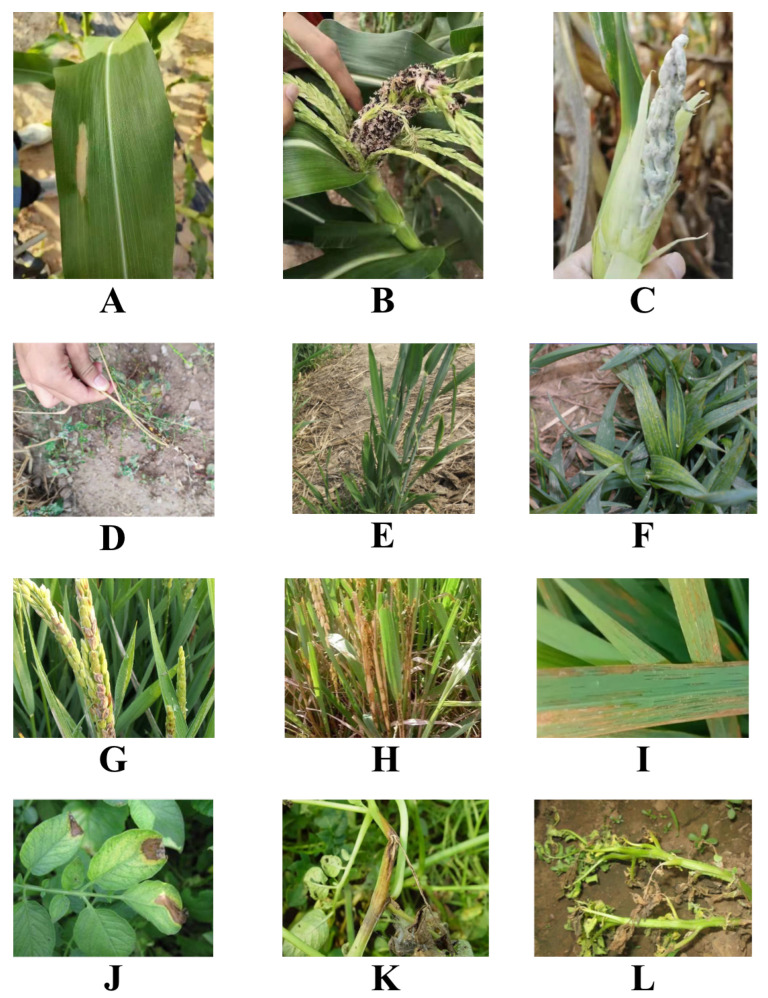
Samples from our dataset. (**A**) macrophthalmia (maize); (**B**) black Sigatoka (maize); (**C**) tumor black powder (maize); (**D**) black Sigatoka (wheat); (**E**) green dwarf (wheat); (**F**) yellow leaf (wheat); (**G**) rice fever (rice); (**H**) stripe blight (rice); (**I**) bacterial streak (rice); (**J**) late blight (potato); (**K**) black shin (potato); (**L**) blight (potato).

The collection devices included Canon Mark 5D, Apple, and Huawei cell phones, as shown in Figure 6.

Due to the diversity of the collection devices, the data were uniformly processed to 224×224 before being used in the model, as discussed in Section 5.2. The crops collected included maize, wheat, rice, and cotton. A total of 16 healthy and diseased crops were collected, and the specific dataset distribution is shown in Table 5.

From Table 5, it is evident that the number of disease datasets varied significantly among crops. Due to the crop maturation cycle, maize images were scarce compared to other crops, and such classes with low data percentages are collectively referred to as weak classes in the following sections. Since machine learning training is highly data-dependent, its core principle is to adjust model parameters through the combination of inputs and outputs, and classes with low data shares may be discriminated against by the model. For instance, if the percentage of weak classes is very low, e.g., only 1%, the model can simply assign input images directly to the strong classes, which can still guarantee an accuracy of 99%. To address this issue, some data enhancement methods were employed to preprocess the model, which are described in detail in Section 5.2.

### 5.2. Dataset Preprocessing

As discussed in Section 5.1, two core problems need to be addressed in dataset preprocessing: unifying the resolution of images acquired by multiple devices to facilitate model processing and data enhancement for weak classes to balance the dataset.

**Figure 7:**
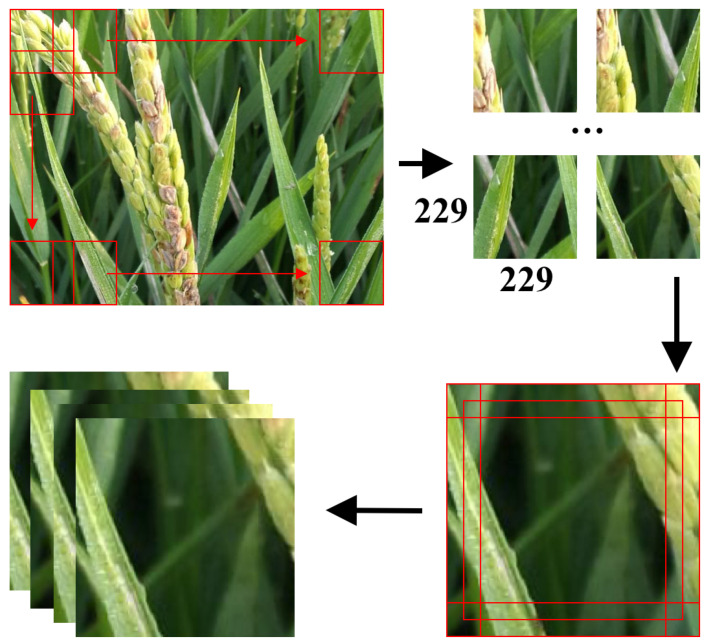
Illustration of the processing of resizing. Given a 229×229 image, we generated five 224 × 224 images using the following procedure: Center crop: Extract a 224 × 224 image from the center of the 229×229 input image. This is done by removing 2 pixels from the left and right borders and 2 pixels from the top and bottom borders of the image. Corner crops: Extract four 224 × 224 images from the four corners of the 229×229 input image. Top-left crop: Remove 5 pixels from the right border and 5 pixels from the bottom border. Top-right crop: Remove 5 pixels from the left border and 5 pixels from the bottom border. Bottom-left crop: Remove 5 pixels from the right border and 5 pixels from the top border. Bottom-right crop: Remove 5 pixels from the left border and 5 pixels from the top border. The red boxes are the sliding windows.

First, the method shown in Figure 7 was employed to process the dataset. The dataset was unified to a 224×224 resolution, which was convenient for model processing.

As illustrated in Figure 7, the original image was scanned with a sliding window of size 229×229, resulting in multiple images. Following that, the 229×229 images were cropped using the method employed in the AlexNet [54] model to generate five 224×224 images, as shown in Figure 7.

At this point, the dataset was resized to 224×224. Next, an adaptive data enhancement strategy was designed. The degree of enhancement was denoted by β, as shown in Equation (1).

(1) $$\beta_{class_{A}}=\frac{1}{\frac{number_{class_{A}}}{number_{all}}}=\frac{number_{all}}{number_{class_{A}}}$$

In this way, the weaker classes received more data augmentation and the corresponding stronger classes, a smaller data augmentation. Eventually, a relatively balanced result could be achieved for each class of the dataset, as shown in Table 6.

**Figure 8:**
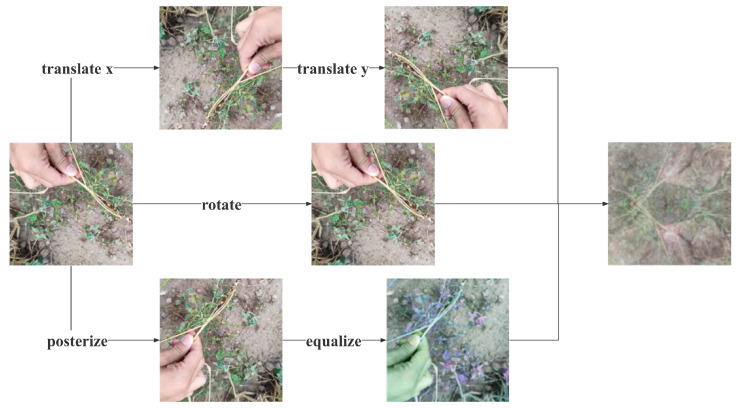
Illustration of the AugMix method. AugMix combines multiple data augmentation operations (such as rotation, translation, shearing, etc.) by mixing their results with different probabilities.

**Figure 9:**
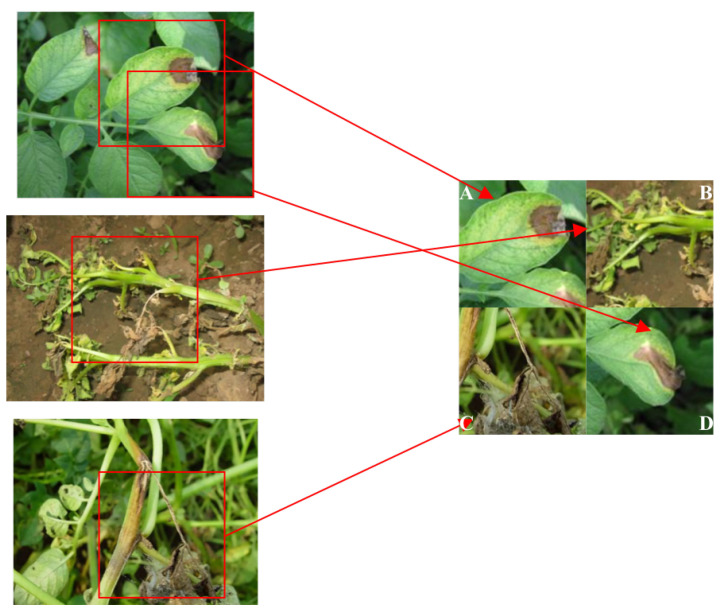
Mosaic schematic illustration: image (A) (top left), image (B) (top right), image (C) (bottom left), image (D) (bottom right) → mosaic (combine the four images into one).

**Figure 10:**
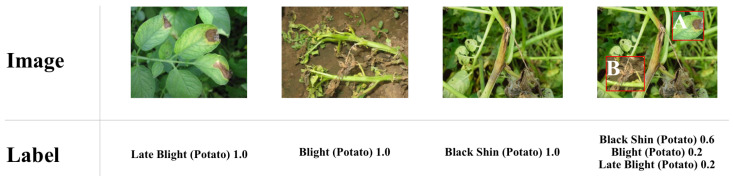
CutMix schematic illustration: image (A) + image (B) (random patch) → CutMix (combines images by pasting a patch from one image to another).

The specific augmentation methods used in this paper included:

1. AugMix [55]: This method first generated three graphs using traditional augmentation methods such as translation, rotation, and equalization. After that, three weights $w_{i}$ were randomly selected using the Dirichlet(1,1,1) distribution, and the weights summed to 1 according to the nature of the Dirichlet distribution. After that, the three chains were summed by the weights $w_{i}$ to obtain $X_{aug}$. Next, $X_{aug}$ and the original $X_{ori}$ were summed by weight using a β(1,1) distribution sampling. The overall process is shown in Figure 8.
2. Mosaic [44]: This method first read four random images from the dataset at a time. After that, it flipped (flipped the original image left and right), scaled (scaled the original image), and changed the color gamut (changed the brightness, saturation, and hue of the original image) of each of the four images. After the operation was completed, the original images were stitched together in a way that the first image was placed on the top left, the second image was placed on the bottom left, the third image was placed on the bottom right, and the fourth image was placed on the top right. Finally, the images were combined, and the frames were assembled. After the four images were placed, we used the matrix to capture the fixed areas of the four images and then stitched them together to form a new image with a series of boxes. The enhancement of this method is shown in Figure 9.
3. CutMix [56]: This method randomly selected a part of the region and filled in the pixel values of the rest of the data in the training set, and the classification labels were mixed and smoothed in a certain proportion, as shown in Figure 10.

### 5.3. Proposed Method

#### 5.3.1. Overall

The proposed method in this paper aimed to improve the performance of the feature extraction network in a target detection framework while maintaining computational efficiency, especially for edge computing scenarios with limited resources, such as agricultural applications. The method consisted of two main components: a structural re-parameterization and a dynamic pruning gate (DPG).

Structural re-parameterization introduces a re-parameterization module to enhance the feature extraction capability of the model. By merging multiple parallel networks during training, the feature extraction capability of multiple networks is integrated without significantly increasing the model parameters. The technique involved decomposing the weights of convolutional layers into fixed base weights and learnable parameters, allowing the network to learn more expressive and diverse feature representations. This results in better performance across various tasks. The method also focuses on improving the model’s robustness to image flipping and rotation.

The DPG module is designed to address the trade-off between accuracy and computational complexity in deep convolutional neural networks. The DPG module predicts the significance of the next convolutional channel and skips the insignificant channels at runtime. Unlike static pruning, which completely removes insignificant channels, dynamic pruning keeps all channels and dynamically skips the insignificant ones during runtime. This enables the model to run smoothly on agricultural computing facilities with low computational power.

**Figure 11:**
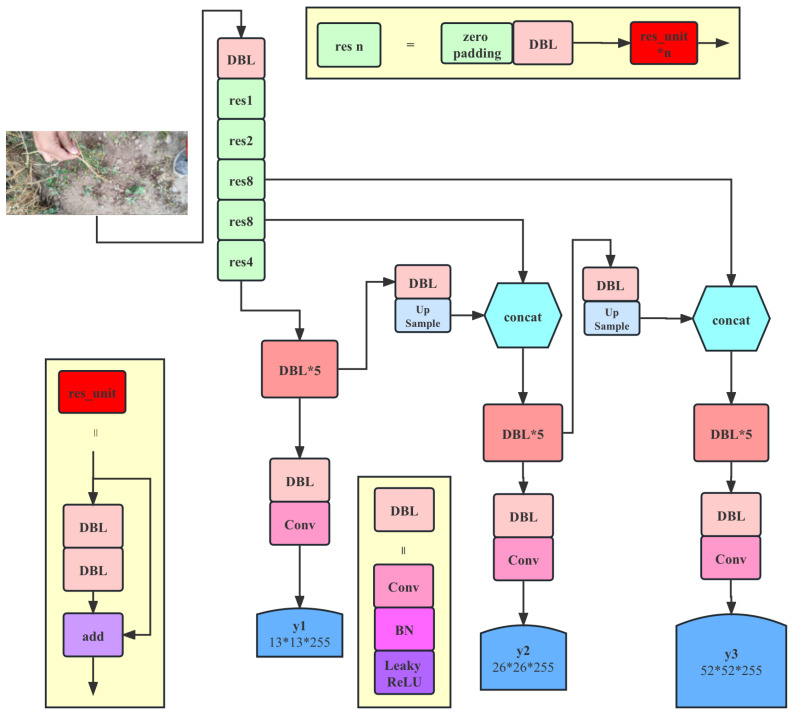
Illustration of proposed method based on detection network. When constructing convolutional neural networks (CNNs), some fundamental building blocks are commonly employed to enhance network performance and training stability. Here are two typical building blocks: (1) DBL (Conv + BN + Leaky ReLU): DBL is a basic module that combines a convolutional layer (Conv), batch normalization (BN), and Leaky ReLU activation function. In DBL, the convolutional layer (Conv) is responsible for extracting local features from the input feature map. Batch normalization (BN) is a regularization technique that accelerates network training and mitigates the issues of vanishing and exploding gradients. Leaky ReLU is a nonlinear activation function with a small negative slope, providing. a certain gradient in the negative region, thereby alleviating the vanishing gradient problem. The DBL module integrates these techniques, rendering network training more stable and enhancing feature extraction capabilities. (2) Res_unit (basic residual block): The residual unit (res_unit) is a fundamental module that utilizes skip connections to effectively address the vanishing and exploding gradient problems in deep networks. A basic residual block consists of two DBL layers, where one DBL layer follows another. There is a skip connection between these two DBL layers, connecting the input directly to the output of the second DBL layer. This skip connection allows gradients to propagate more easily within the network, making deep networks easier to train.

The methodological structure of this paper is shown in Figure 11.

**Figure 12:**
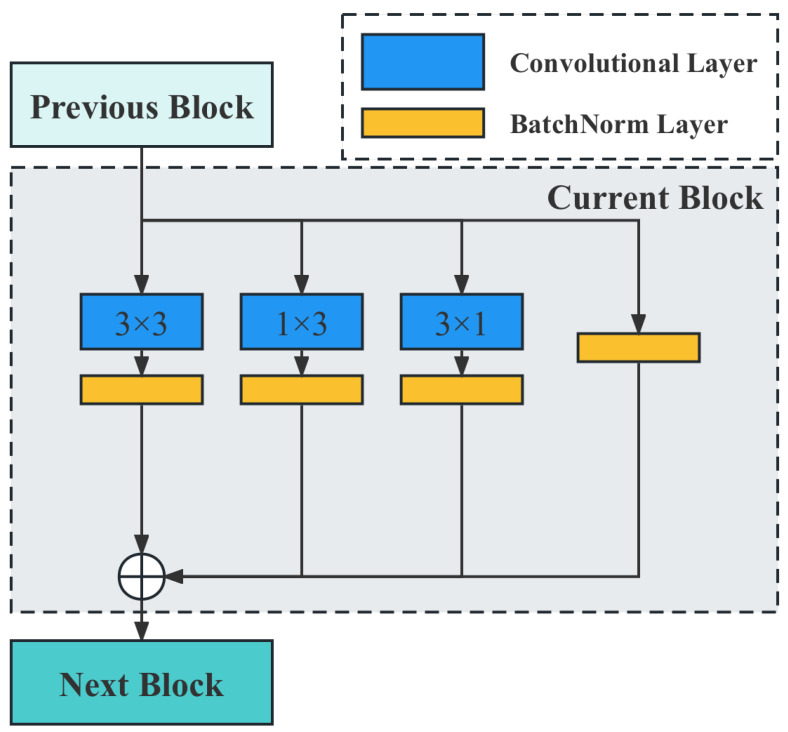
Illustration of the basic block used in our neural network. The gray dashed box represents the basic block used to construct the model in this paper. The blue blocks represent the convolutional layers, while the orange blocks represent the batch normalization (BN) layers. When the output from the previous layer enters the current block, it is processed through four separate branches. After undergoing the processing illustrated in the figure, the outputs of these branches are concatenated. The resulting output is then fed into the next block.

As seen in Figure 11, the proposed method is based on the target detection network, which consists of two parts: the feature extraction network and the target detection network. The performance primarily depends on the feature extraction network. Therefore, effectively improving the performance of the feature extraction network becomes the main consideration of the model design. The current feature extraction network is essentially modularized, meaning the entire network is quickly built by reusing well-designed basic blocks. The basic block used in this paper is shown in Figure 12.

From Figure 12, it is evident that in order to enhance the feature extraction capability of the model, a re-parameterization module is introduced. By merging multiple parallel networks during training, the feature extraction capability of multiple networks can be integrated without significantly increasing the model parameters. Additionally, a dynamic pruning gate module is innovatively proposed, which dynamically predicts the significance of the next convolutional channel and skips the insignificant channels when the program is run. In this way, dynamic pruning enables the model to run smoothly on agricultural computing facilities with low computational power.

#### 5.3.2. Structural Re-Parameterization

Model parameters primarily refer to the learned parameters and other parameters obtained during the training process, such as the mean and standard deviation obtained cumulatively by the batchnorm layer. Then, a set of parameters and a structure are in one-to-one correspondence. For instance, if there is no nonlinearity between two fully connected layers *a* and *b*, they can be converted into a fully connected layer *c*. Let the parameters of these two fully connected layers be matrices *A* and *B*, and the input is *x*. The output is y=B(Ax). A matrix C=BA can be constructed, then y=B(Ax)=Cx. Then, *C* is the parameter of the fully connected layer obtained. Then, the parameter AB corresponds to the structure ab, and the parameter *C* corresponds to the structure *c*.

**Figure 13:**
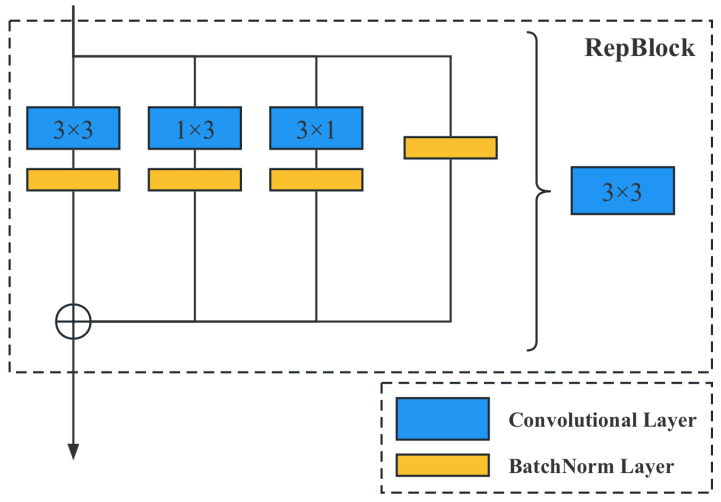
Illustration of the re-parameterization method. RepBlock, short for re-parameterization block, is a novel technique designed to improve the performance of convolutional neural networks (CNNs) by re-parameterizing the weights of convolutional layers. The main idea behind RepBlock is to introduce additional parameters into the network that allow for a more efficient feature extraction and adaptability in the learning process. The RepBlock technique enhances the performance of CNNs by re-parameterizing the weights of convolutional layers. By decomposing the weights into fixed base weights and learnable parameters, the network can learn more expressive and diverse feature representations, ultimately leading to a better performance in various tasks.

Structural re-parameterization essentially means constructing a set of structures (typically for training) and equivalently converting their parameters to another set of parameters (typically for inference or deployment), thus equivalently converting this set of structures to another set of structures. In a realistic scenario, where training resources are generally relatively abundant, the focus is on inference-time overhead and performance. Therefore, larger training-time structures with good properties (a higher accuracy or other useful properties, such as sparsity) are desired, and smaller converted inference-time structures that retain such properties are also desired. In this way, the training-time structure corresponds to a set of parameters, and the desired inference-time structure corresponds to another set of parameters; the former structure can be equivalently converted to the latter as long as the parameters of the former can be equivalently converted to the latter. Structure *A* corresponds to a set of parameters *X*, and structure *B* corresponds to a set of parameters *Y*. If *X* can be equivalently converted to *Y*, structure *A* can be equivalently converted to *B*. Figure 13 demonstrates how this method can be applied to convolutional neural networks.

During the training phase, Reparam(K×K)=(K×K)+(1×K)+(K×1) denotes replacing a K×K convolution with the sum of three parallel branches (K×K,1×K,K×1). As illustrated in Figure 13, a 3×3 convolution can be obtained by convolving 3×3+1×3+3×1; finally, the results of these three convolutional layers are fused to obtain the convolutional layer output. It is important to note that the 3×3, 1×3, and 3×1 convolutions of the trained model are fused first, and then the new model is derived and ultimately used.

**Figure 14:**
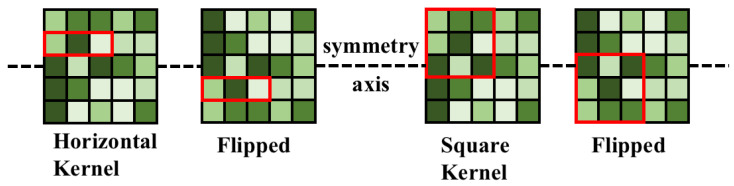
Asymmetric convolution is more robust to up-and-down flips than square convolution. The red boxes indicate the paired features.

In addition, the asymmetric convolution is more robust to up-and-down flips than the square N×N convolution of. The model in this paper, as shown in Figure 13, focused on improving the robustness of the model to image flipping and rotation. As shown in Figure 14, when the 1×3 convolution kernel is introduced in the training phase, the trained 1×3 convolution kernel can still extract the correct features even if the input image is flipped downward in the validation phase.

In Figure 14, the two red rectangles are the image features extracted before and after the image flip operation and are still the same at the same position of the input image. For the square-shaped convolution, the extracted features are different.

#### 5.3.3. Dynamic Pruning Gate

**Figure 15:**
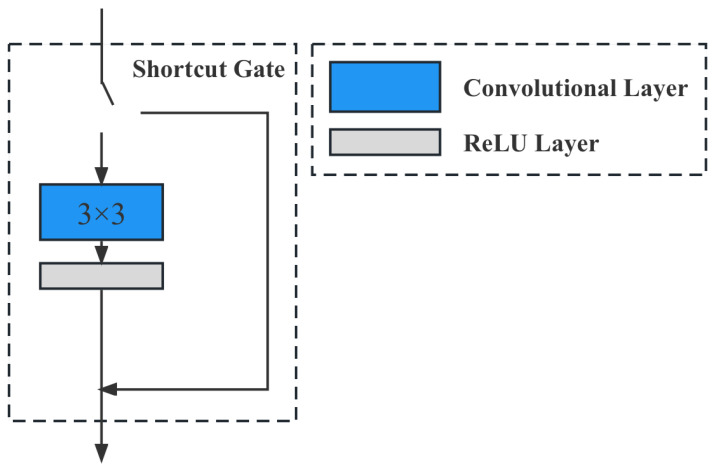
Illustration of the dynamic pruning gate (DPG) module. The DPG module is a technique designed to address the trade-off between accuracy and computational complexity in deep convolutional neural networks, particularly in edge-computing scenarios with limited resources, such as agricultural applications. The main goal of DPG is to improve the efficiency of convolutional networks for data feature extraction without significantly increasing computation and memory requirements.

It is a generally accepted fact in deep learning that to improve the accuracy of deep convolutional neural networks inevitably causes an increase in computation and memory. However, in agricultural scenarios, computing devices deployed in edge-computing scenarios generally have poor computing power. Therefore, it is important to design a network structure to improve the efficiency of convolutional networks for data feature extraction. Therefore, this paper proposes a dynamic pruning gate (DPG), whose structure is shown in Figure 15.

From Figure 15, we can see that this module predicts the significance of the next convolutional channel and skips the insignificant channels when the program is run. Unlike static pruning, which completely cuts out the insignificant channels, dynamic pruning keeps all channels and dynamically skips the insignificant channels during the program runtime. In addition, the network with the DPG method is still trained with the traditional SGD, so DPG is adapted to various SOTA CNN models.

#### 5.3.4. Loss Function

For a given pair of image sequences $\left(s_{i},s_{j}\right)$, each sequence is processed by the network to obtain the sequence feature vectors as $v_{i}=R\left(s_{i}\right)$ and $v_{j}=R\left(s_{j}\right)$. Its loss function is defined as follows:

(2) $$Loss=E\left(R\left(s_{1}\right),R\left(s_{2}\right)\right)+I\left(R\left(s_{1}\right)\right)\;+\;I\left(R\left(s_{2}\right)\right)$$

*E* denotes the contract loss, which is defined as follows:

(3) $$E\left(v_{i},v_{j}\right)=\left\{\begin{array}{c} \frac{1}{2}\left|\right|v_{i}-v_{j}{\left|\right|}^{2},\;i=j\\ \frac{1}{2}[max(m-\left|\right|v_{i}-v_{j}{\left||,0)\right]}^{2},\;i\neq j \end{array}\right.$$

*I* denotes the identity loss, which is defined as follows:

(4) $$I\left(v\right)=P\left(q=c|v\right)=\frac{exp(W_{c}v)}{\sum_{k}exp\left(W_{k}v\right)}$$

### 5.4. Evaluation Metrics

In this paper, precision, recall, and mean average precision (mAP) were utilized as evaluation metrics. Equations (5) and (6) show the formulae for the precision and recall, respectively.

(5) $$Precision=\frac{TP}{TP\;+\;FP}$$

(6) $$Recall=\frac{TP}{TP+FN}$$

Since the method used in this paper was based on multilabel images, the mAP evaluation index for multilabel classification was used, and the AP expressed below measured the strengths and weaknesses of the training model in each category. The mAP integrates the strengths and weaknesses of all categories based on the AP to obtain comprehensive evaluation results. The mAP was calculated as shown in Equation (7).

(7) $$mAP=\frac{\sum_{i=1}^{k}AP_{i}}{k}$$

The AP in the above equation was calculated as follows:

(8) $$AP=\sum_{i=1}^{n-1}\left(r_{i\;+\;1}-r_{i}\right)Precision_{inter}\left(r_{i}\;+\;1\right)$$

$r_{1},r_{2},\cdots ,r_{n}$ are the recall values corresponding to the first interpolation of the precision interpolation segment in ascending order.

## 6. Conclusions

Timely and accurate detection of plant disease in the rearing environment is an important research topic. A dynamic pruning-based method for automatic detection of lesion patterns in low-computing scenarios was proposed. The main contributions of this research work include the following:

1. A re-parameterization method was proposed to improve the boosting accuracy of convolutional neural networks.
2. A dynamic pruning gate was proposed to dynamically control the network structure so that it could run on hardware platforms with significant differences in computing power.
3. The theoretical model based on this study was implemented, and the development of the application program was completed.

The experimental results showed that the model could run under a variety of computing platforms, including GPU platforms with high computational performance and mobile terminal platforms with very low computing power, and its inference speed, 58 FPS, was faster than that of other mainstream models. In terms of model accuracy, we enhanced the subclasses with low detection accuracy by data augmentation methods and verified them by ablation experiments. The model was finally able to achieve a 0.94 precision. In order to make full use of the above experimental results, we developed an application based on the model so that the model could be effectively deployed in real agricultural scenarios.

## Figures and Tables

**Table 1:** The table summarizes the detection results for different object detection models in terms of mAP@75, mAP@50, recall, and precision. The models compared include Faster RCNN, SSD, YOLO v3, YOLO v4, and the proposed method.

Model	mAP@75	mAP@50	Recall	Precision
Faster RCNN [42]	0.46	0.61	0.41	0.58
SSD [43]	0.52	0.73	0.48	0.57
YOLO v3 [33]	0.67	0.79	0.65	0.73
YOLO v4 [44]	0.69	0.78	0.66	0.79
Ours	0.78	0.92	0.73	0.94

**Table 2:** Speeds (frames per second) of different detection models on different platforms. In this table, only the model in this paper could run locally on the Huawei P40 and achieved an inference speed of 17 FPS, while all other models were unable to perform inference under the local computing power and memory limitations of the Huawei P40.

Model	RTX 3080 GPU	PC	Jetson Nano	Huawei P40
Faster RCNN [42]	12	8	5	-
SSD [43]	21	17	9	-
YOLO v3 [33]	35	28	19	-
YOLO v4 [44]	33	29	17	-
Ours	58	49	42	17

**Table 3:** Detection results on other datasets using different models.

Model	Kaggle	Plantdoc
Faster RCNN [42]	0.54 [26]	0.38 [22]
SSD [43]	0.64 [26]	0.38 [22]
YOLO v3 [33]	0.58 [26]	0.39 [22]
YOLO v4 [44]	0.63 [26]	0.38 [22]
Ours	0.66	0.48

**Table 4:** Detection results on more datasets.

Research Topic	Metric	Method	Result	FPS
Wheat head detection	mAP	[26]	0.6756 [26]	-
		Ours	0.6748	58
Maize disease detection	Accuracy	[24]	97.41% [24]	-
		Ours (backbone + softmax)	95.38%	49
Apple flower detection	mAP	[25]	0.9743 [25]	-
		Ours	0.9438	63
Leaf disease detection	mAP	[22]	0.503 [22]	-
		Ours	0.528	58

**Table 5:** The dataset used in this study consisted of images from four different crops: maize, wheat, rice, and potato. This table provides a detailed overview of the distribution of the dataset, including the number and proportion of images for each crop and disease. For maize, there were 1291 healthy images (8.46% of the dataset), 283 images of macrophthalmia (1.86%), 197 images of black Sigatoka (1.29%), and 84 images of tumor black powder (0.55%). In the wheat category, there were 2013 healthy images (13.19%), 397 images of black Sigatoka (2.60%), 513 images of green dwarf (3.36%), and 523 images of yellow leaf (3.43%). For rice, the dataset contained 4843 healthy images (31.73%), 731 images of rice fever (4.79%), 293 images of stripe blight (1.92%), and 423 images of bacterial streak (2.77%). Lastly, in the potato category, there were 2382 healthy images (15.61%), 472 images of late blight (3.09%), 581 images of black shin (3.81%), and 238 images of blight (1.56%).

Crop	Disease	Number	Proportion
Maize	Healthy	1291	8.46%
	Macrophthalmia	283	1.86%
	Black Sigatoka	197	1.29%
	Tumor black powder	84	0.55%
Wheat	Healthy	2013	13.19%
	Black Sigatoka	397	2.60%
	Green dwarf	513	3.36%
	Yellow leaf	523	3.43%
Rice	Healthy	4843	31.73%
	Rice fever	731	4.79%
	Stripe blight	293	1.92%
	Bacterial streak	423	2.77%
Potato	Healthy	2382	15.61%
	Late blight	472	3.09%
	Black shin	581	3.81%
	Blight	238	1.56%

**Table 6:** After applying data augmentation techniques, the distribution of the dataset was balanced across all crops and diseases. This table provides a detailed overview of the distribution of the augmented dataset, including the number and proportion of images for each crop and disease. For maize, the dataset then consisted of 15,260 healthy images (6.25% of the dataset), 15,215 images of macrophthalmia (6.23%), 15,271 images of black Sigatoka (6.25%), and 15,272 images of tumor black powder (6.26%). In the wheat category, there were 15,261 healthy images (6.25%), 15,269 images of black Sigatoka (6.25%), 15,267 images of green dwarf (6.25%), and 15,247 images of yellow leaf (6.24%). For rice, the augmented dataset contained 15,263 healthy images (6.25%), 15,260 images of rice fever (6.25%), 15,260 images of stripe blight (6.25%), and 15,270 images of bacterial streak (6.25%). Lastly, in the potato category, there were 15,259 healthy images (6.25%), 15,275 images of late blight (6.26%), 15,249 images of black shin (6.25%), and 15,256 images of blight (6.25%). The data augmentation process effectively balanced the dataset by ensuring that each disease category had a similar number of images, which led to a more robust and reliable model.

Crop	Disease	Number	Proportion
Maize	Healthy	15,260	6.25%
	Macrophthalmia	15,215	6.23%
	Black Sigatoka	15,271	6.25%
	Tumor black powder	15,272	6.26%
Wheat	Healthy	15,261	6.25%
	Black Sigatoka	15,269	6.25%
	Green dwarf	15,267	6.25%
	Yellow leaf	15,247	6.24%
Rice	Healthy	15,263	6.25%
	Rice fever	15,260	6.25%
	Stripe blight	15,260	6.25%
	Bacterial streak	15,270	6.25%
Potato	Healthy	15,259	6.25%
	Late blight	15,275	6.26%
	Black shin	15,249	6.25%
	Blight	15,256	6.25%
